# Exploring Older Adults’ Perspectives on Digital Home Care Interventions and Home Modifications: Focus Group Study

**DOI:** 10.2196/52834

**Published:** 2024-12-13

**Authors:** Mohamed-Amine Choukou, Jasem Banihani, Sarah Azizkhani

**Affiliations:** 1 Department of Occupational Therapy College of Rehabilitation Sciences, Rady Faculty of Health Sciences University of Manitoba Winnipeg, MB Canada; 2 Centre on Aging, University of Manitoba Winnipeg, MB Canada; 3 Biomedical Engineering program University of Manitoba Winnipeg, MB Canada

**Keywords:** agetech, attitude, opinion, perception, perspective, home based, community based, research, strategic planning, gerontechnology, geriatric, older adults, aging, co-construction, workshop, inductive analysis, development, aging-in-place, independent

## Abstract

**Background:**

Emerging gerontechnology seeks to enable older adults (OAs) to remain independently and safely in their homes by connecting to health and social support and services. There are increasing attempts to develop gerontechnology, but successful implementations are more likely limited because of the uncertainty of developers about the needs and priorities of OAs. As the global population ages, the challenges faced by older OAs in maintaining independence and well-being within their homes have become increasingly important. With the proportion of OAs expected to triple by 2068, addressing the needs of this demographic has become a pressing social and public health priority. OAs often encounter various challenges related to physical, cognitive, and social well-being, including reduced mobility, memory impairments, and social isolation, which can compromise their ability to age in place and maintain a high quality of life.

**Objective:**

The goals of this qualitative research study are to (1) determine the best strategies for promoting aging well in the community with the support of gerontechnology, (2) establish the top priorities for implementing gerontechnology with OAs and their families, and (3) create a road map for the creation and application of gerontechnology for aging well in Manitoba.

**Methods:**

A total of 14 OAs participated in a qualitative research study conducted through a coconstruction workshop format, including a presentation of novel research facilities and a demonstration of research and development products. This activity was followed by an interactive discussion focused on revisiting the ongoing research and innovation programs and planning for a new research and innovation agenda. The workshop contents, notes, and recorded conversation underwent a data-driven inductive analysis.

**Results:**

Emerging themes included home design, accessibility, and safety for OAs, particularly those with memory impairments. The participants also underlined the need for digital reminders and ambient technologies in current homes as a priority. Participants stressed the importance of including OAs in gerontechnology development programs and the need to consider dignity and independence as the guiding values for future research.

**Conclusions:**

This study presents a tentative road map for the development of gerontechnology in Manitoba. The main principles of our road map are the inclusion of OAs as early as possible in gerontechnology development and the prioritization of independence and dignity. Applying these principles would contribute to combatting digital ageism and the marginalization of OAs in technology development because of the perceived lack of technological skills and the stereotypes associated with this presumption.

## Introduction

As the average age rises due to greater life expectancy, health care systems face a growing number of older adults (OAs) who require services to maintain at-home independence [1-4]. According to a Statistics Canada report published in September 2019, the number of people aged 80 years and older is expected to triple by 2068 [5]. This significant demographic transition will have far-reaching societal consequences, including increased health care strain [2,3]. OAs’ health and wellness needs are complex and multifaceted. Aging-related processes can lead OAs to experience a range of physical, cognitive, and social challenges [6] along with mood disorders in particular among those with comorbidities. Physically, they might face issues like reduced mobility, slower walking speed, falls, frailty, and difficulties in basic and instrumental daily activities, all contributing to a diminished quality of life. Cognitively, OAs may encounter problems such as memory lapses, hearing loss, cataracts, refractive errors, presbyopia, reduced sensory and vestibular functions, and increased agitation and apathy [7]. In terms of mental illness among people with comorbidities, a recent paper presenting data from over 24,000 individual surveys showed that having both heart disease and cancer was independently associated with serious mental illnesses and that patients with a combined diagnosis of cancer and heart disease were significantly more likely to report serious mental illnesses than those with either diagnosis alone. Overall, these challenges often result in behavioral disturbances and limited social engagement. Gerontechnology offers potential solutions to these challenges. Gerontechnology is defined as “an interdisciplinary field that links existing and developing technologies to the aspirations and needs of aging and aged adults… that helps support ‘successful aging,’ is organized according to the WHO definition of health, and is a response to the combination of the aging of society and rapidly emerging new technologies” [8]. Effective strategies are thus needed to promote aging well. Halaweh et al [9] identified the contributing factors to aging well as having a physically active lifestyle, participating in social and leisure activities, following healthy eating habits, having a purpose in life, and staying intellectually engaged [9]. One of the determinants of aging well is to age in place as long as possible [4]. This strategy for aging well requires home adaptations and technology upgrades and adopting technologies to support aging at home.

OAs are currently aging in place with assisted living, supportive housing, and home care solutions. Emerging technologies such as the Internet of Things, which describes the network of physical objects that are embedded with sensors, software, and other technologies for the purpose of connecting and exchanging data with other devices and systems over the internet, artificial intelligence, which is the ability of a computer or computer-controlled robot to perform tasks that are commonly associated with the intellectual processes characteristic of humans, sensors, which are devices that detect and respond to some type of input from the physical environment, cloud computing, which is the delivery of computing services (including servers, storage, databases, networking, software, analytics, and intelligence) over the internet to offer faster innovation, flexible resources and economies of scale, wireless communication technologies, and assistive robotics have aided in the development of various gerontechnology such as wearables and ambient assisted living approaches to help OAs live independently and safely in their home [4]. Ambient assisted living comprises methods, concepts, systems, products as well as services, which support the everyday life of OAs and people with disabilities with situation-dependent and unobtrusive support [10]. In a recent study, we found that both older and younger adults see technology for assisted living as promising for enhancing OAs’ independence at home but share concerns about privacy and data security [11]. These technologies motivate OAs to engage in activities that uphold their physical and mental health, eventually enhancing their quality of life. The need for telehealth has grown even more during and after the COVID-19 pandemic as telehealth technologies provide users with direct access to health care services without leaving their homes [12]. Many telehealth solutions allow the user to videoconference with health care professionals [13] and obtain health-related information through mobile devices, such as smartphones and wearable sensors [12,14]. Among telehealth solutions, ambient assisting living technology (AALT) is the technology that seems adequately dedicated to meeting the OAs’ needs of aging in place. AALT is primarily based on the Internet of Things concept and includes smart physical objects and other devices capable of collecting patient health information and sharing it with care providers and caregivers as needed [6]. An example of AALT is a smart pill dispenser in an OAs’ home that could remind them to take their medications on time and alert caregivers if a dose is missed [15]. This type of gerontechnology is undoubtedly a promising approach to promoting ageing in place and aging well [12], but the needs for those technologies and their clinical relevance remain questionable. For example, a plethora of AALT is reported in the literature, yet the needs, priorities, and benefits of using existing AALT for aging well remain understudied [16]. The demand for and ultimate use of gerontechnology has yet to be finetuned with the end users. The “end-users” term means not only OAs but includes all the stakeholders such as clinicians, families, and technology providers. A theoretical framework is needed to guide the understanding and navigation of the AALT ecosystem. There is a lack of clear road maps and frameworks to support the design of gerontechnology. Many theoretical frameworks have been proposed, such as the Diamond framework [17]. Although beneficial for consultation and development, existing theoretical frameworks are not oriented toward direct research and development to fulfill specific profiles’ health and well-being demands but rather holistic. The deployment of such a framework necessitates its integration with local needs and recommendations, which turns the framework’s directions into concrete development activity. Various empirical studies explored the relevance and efficacy of telehealth to support the health of OAs. For example, technology can help assess OAs’ loneliness and social isolation [18]. Another study on smart home deployment demonstrated potential in managing chronic diseases by controlling exacerbations and enhancing patient safety, especially for older individuals with cognitive impairment [19]. A recent systematic review on gerontechnology for aging in place [20] found limited evidence of efficacy for most technologies aimed at supporting OAs and their caregivers.

Ambient sensing research seems to lack in-situ implementation and most of the research centers around validating AALT in simulated environments (laboratory settings) [13,17-20], while testing in naturalistic contexts is highly needed. In addition, the lack of user perspective in ambient sensing research raises questions about the usability of these technologies for home care. Indeed, there are gaps in the AALT field between the users’ wants and needs regarding health and wellness and the current research and development. There is an increasing need to comprehend the demands and priorities of OAs regarding gerontechnology to advance this area of applied research. Recent literature recommends that OAs from various backgrounds and health statuses be partners in the research program [21-26]. OAs know better about their current aging matters and are interested in learning new technologies [27] as they are well positioned to know their needs but do not necessarily understand the goals, functionalities, and benefits of each gerontechnology. To improve acceptability and dissemination, literature [20] recommends incorporating user values and preferences, cocreating with end users, designing user-friendly technologies, and providing adequate training. This study aims to portray the perspectives of 14 OAs on gerontechnology for aging well based on current literature and research. We gathered a group of OAs from various socioeconomic backgrounds to take part in a qualitative research study conducted through a coconstruction workshop format to determine the best strategies for promoting aging well in the community with the support of gerontechnology, establish the top priorities for implementing gerontechnology with OAs and their families, and create a road map for the creation and application of gerontechnology for aging well in the province of Manitoba.

## Methods

### Study Type

This qualitative research study is based on a 3-hour coconstruction workshop with OAs living in the community in Manitoba who were invited by email distributed by the Manitoba Association of Senior Communities and among the principal investigator’s database of research participants. This research is reported in accordance with the consolidated criteria for reporting qualitative research (COREQ) checklist [28].

### Participants

A total of 14 OAs participated in the coconstruction workshop including 10 females and 4 males. The participants had a mean age of 70.9 (SD 2.7) years, with ages ranging from 67 to 76 years. One used a walker, and one used a wheelchair for mobility. The rest were able to walk with no assistance. In total, 13 participants speak English as their first language. One participant speaks English as their second language and had no issue participating in the discussion.

#### Workshop Procedures

We organized a 3-hour workshop at the local Health Sciences Centre led by the principal investigator of the study [MAC]. One week before the gathering day, we shared information by email with our participants to introduce them to our facility as an introduction to the gerontechnology we developed and our vision for supporting aging well in the community. The information shared includes descriptive text and links to our video. The workshop consists of 4 steps, each lasting 1 hour.

#### First Step: Discovery Phase

**Figure 1:**
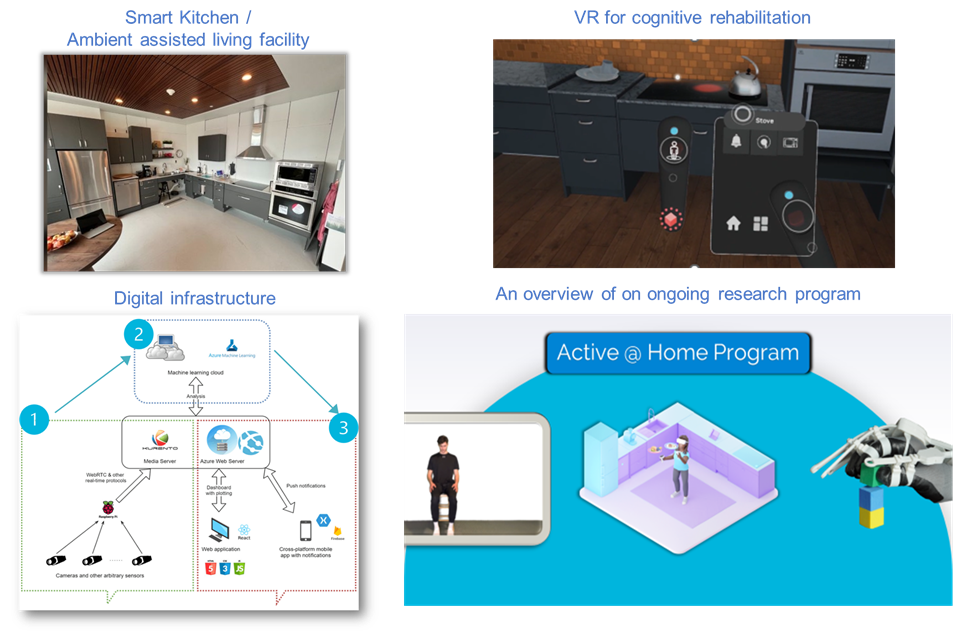
An overview of the gerontechnology presented to workshop participants. VR: virtual reality.

We dedicated the first hour to discovering and discussing the ongoing research and innovation agendas, which started by presenting the gerontechnology available in our facility. During this time, all the participants were in the ambient assisted living facility and were given ample time to interact with the equipment and ask questions interactively to one of the 3 authors of this paper ensuring clarity on all facility concepts (JBH, SG, and MAC). The questions were gathered individually from each participant, but responses were provided verbally to the entire group. The facility tour included exploring the following conceived technology or concepts in progress (Figure 1):

- An ambient assisted living facility [29]. The smart kitchen shown in Figure 1 showcases the potential for integration of high-adjustable pantries, storage spaces, and preparation sink and cooking areas [29]. The cabinetry is controlled by an app on a tablet. VR technology illustrates the potential for task-oriented training in a virtual environment to support neurorehabilitation in a health care facility or home [30]. Our e-prototype of digital infrastructure allows for ambient sensor-based data integration. We guided the participants to our ambient assisted living facility to show them our facility and discuss potential integration in current homes. First, we showed them the smart kitchen. In this kitchen, we indicated the height-adjustable counters and table and kitchen appliances that can be controlled using a mobile app. Then, we showed them 2 bathrooms designed to demonstrate flexible options for accessibility and a bedroom where appliances and electronics can be controlled independently through voice or remote switches.
- A virtual reality (VR) environment [30] and telerehabilitation app [30]. Figure 1 also shows the Active at Home program, an ongoing telerehabilitation program for people with stroke that incorporates our technologies, namely an app on a tablet for neuromotor training, the VR environment for cognitive training, and the hand telerehabilitation platform. We showed our participants the technologies developed for the active at-home program including an app on a tablet and a simulated VR environment that provides physical and cognitive training activities, respectively, and the iManus smart glove which is a bilateral smart telerehabilitation glove, outfitted with multiple sensors, attached to a patient’s hand to allow a continuous and safe practice, at their own pace and in the comfort of their home. The OAs did not test the technologies but watched videos showing how the system works in the hand of real patients.
- Telepresence, which refers to the technology that allows individuals to feel as if they are physically present or immersed in a remote location, like videoconferencing.
- Telemonitoring e-prototype, which refers to remotely tracking and monitoring various parameters, such as health data or environmental conditions.

#### Second Step: Chat Time and General Discussion About the Concepts Showcased

We spent the second hour informally discussing the concepts presented and asking the researchers questions if needed. The researchers were present in the room for lunchtime and only took notes of relevant topics when invited to interact with one of the naturally formed subgroups.

#### Third Step: Coconstruction Session

We then used the third hour to discuss and advise on planning new research and innovation agendas in the province. This last hour took a focus group form where participants were asked to discuss their needs and priorities for aging well in Manitoba. The quasi-focus group discussion centered around the availability and accessibility of technology, cost, impairments hindering the use of technology, technology adaptation for the aging population, and accessibility and safety of the home environment.

### Data Collection and Data Analysis

The coconstruction session was voice-recorded, and the contents underwent a data-driven inductive analysis [31] and the procedure followed the recommendation by Braun and Clarke [32]. Two reviewers, JBH and SA, independently analyzed the data, allowing themes to emerge naturally. The authors then iteratively discussed and organized these themes without imposing a preconceived framework, aiming to uncover inherent patterns in the data. Rather than discarding any themes, they integrated them into broader, cohesive themes. Emerging themes were then presented in this paper as a tentative road map developed with the support of older Manitobans to reinforce the meaningful engagement of OAs in research and implementation projects and their contribution to a successful gerontechnology research agenda in Manitoba.

### Ethical Considerations

This research was approved by the University of Manitoba Health Research Ethics Board (HREB H2022:301 [HS25684]). Informed consent was obtained from each participant by email before data collection. All audio recordings were transcribed verbatim and deidentified. Similarly, data collected during the coconstruction workshop, including researchers’ notes, was deidentified after the session. Participants were not compensated.

## Results

### Overview

The findings from this study were grouped into 4 categories per home space characteristics (“Home design, accessibility, and safety”), OAs’ health needs and supporting technology (“Memory impairment and the role of ambient technologies and reminders”), OAs’ needs and desires (“Dignity and independence as the guiding values for future research”) and the role of OAs in research beyond being research participants (“Role of older adults in future research”).

### Home Design, Accessibility, and Safety

Most of the discussion centered around the most hazardous location in the house and the strategies to overcome the issues of design, accessibility, and safety. Participants consistently and repeatedly discussed the hazardous events that usually happen in the kitchen, washroom, and bedroom. They particularly highlighted the significance of kitchen design in terms of accessibility, safety, and functionality. When discussing the smart kitchen design, OA1 emphasized the height-adjustable cabinetry and working spaces and expressed a positive impression about this feature, such as

I thought the kitchen was well designed. I think there is much potential for the idea of the cabinets and especially the sink and everything going up and down

The sink is indeed height adjustable and wheelchair accessible making it more ergonomically suitable for people of different heights and mobility profiles, such as those who use a walker or wheelchair. However, OA4 raised concerns about potential hazards in the kitchen and the difficulties individuals with hand impairments may face thinking of their mother, who was not in the workshop. They mentioned their mother’s struggles with holding heavy objects and dropping things. This realization led them to emphasize the importance of kitchen design, considering the needs of people with different abilities in terms of balance and stability while performing standing and seated cooking tasks. OA1, supported by OA2 and OA3 proposed a potential solution by adding a small extension on the countertop edge to prevent items from falling off while maintaining functionality.

OA10 brought attention to the needs of OAs who use walkers in the kitchen. They shared that their galley kitchen worked well, as they could touch both sides. However, they mentioned having friends who also used walkers while working in the kitchen. OA10 suggested including horizontal bars facing the kitchen user and coming out of the counters, which function as handles providing stability in addition to having walkers. OA1 and OA7 further developed this concept by exploring retractable stabilizers that could fold to let users transition between adjacent kitchen workstations. Besides, OA10 expressed the need for seating options in various kitchen areas. They mentioned the importance of having a sturdy shelf where they could sit and perform tasks like chopping vegetables. They explained that sitting in the kitchen was beneficial for conserving energy, particularly for individuals experiencing fatigue due to multiple sclerosis. OA4 raised a concern about the accessibility of the refrigerator, particularly for people using wheelchairs. She questioned how someone in a wheelchair could reach the top shelf of the large refrigerator in the kitchen. This underlined the importance of accessible design considerations that satisfy wheelchair users' requirement to reach objects overhead safely. In the context of refrigerators, the comment raised by OA4 is more of an appliance accessibility issue that was not part of the current project. Research is needed to identify and recommend accessible refrigerators or to support the development of novel accessible kitchen appliances.

The bedroom and bathroom were also discussed repeatedly. During the conversation, accessibility and usability came up several times to the bedroom and bathroom. OA11 expressed concerns about the existing bathroom design in the ambient assisted living facility and suggested that having small steps instead of ramps would be more convenient for them due to osteoarthritis. OA1 stated,

In the smart bathroom, there's a ramp when you first walk in, but it's a very small and gradual one. However, I have a problem with my ankles due to osteoarthritis, and I can't bend my feet up that much. So, when it comes to ramps, they cause shockwaves to my ankle and are uncomfortable for me. Someone was talking about installing ramps around the house, but for me personally, it's better to have a few steps to climb rather than a ramp. Ramps may be easier for some people, but for me, they're not ideal because of my ankle issues.

### Memory Impairment and the Role of Ambient Technologies and Reminders

We presented a prototype of a smart mirror including the hardware and in-house developed algorithms and discussed its potential integration with reminders for daily activities, whether in the bathroom or elsewhere in the house. For example, it could be positioned near the door to remind people about their wallets and keys before they leave the house. OA10 mentioned her memory problems and highlighted the usefulness of a mirror by the door to remind her to grab essential items like keys, phones, and wallets before leaving the house. She shared that her husband had supplemented the mirror with a checklist to assist her further. OA14 (OA3’s wife) added that having another mirror in the bathroom to remind her to take her pills would be helpful. OA1 reiterated the importance of these reminders for individuals with memory issues, expressing their reliance on checklists that could be available on today’s smart mirrors. OA2, OA9, and OA13 joined the conversation and suggested having 2 mirrors, 1 in the bathroom to remind them to take their pills and 1 by the door to remind them of their keys and phone. They emphasized that while it may be a simple solution, it effectively works for them.

OA6 suggested the idea of motion detector lights, particularly in the bedroom and bathroom, to assist OAs who may forget to turn on the lights when they get up at night. This would help reduce the risk of falls for every kind of resident. Another participant (OA12) emphasized the importance of personalized design based on the specific needs of OAs. They proposed prioritizing certain areas, such as the bedroom or bathroom, over others to ensure their functionality and accessibility specifically for each user. Furthermore, OA9 and OA13 mentioned the convenience of voice activation in the bedroom for operating various household devices and everywhere in the house through robots and tablets. They expressed excitement about the potential of this technology, as it would allow them to control lights and appliances without having to leave the bed. They believed it could significantly enhance their daily routines, especially during periods of weakness such as when someone is sick.

### Dignity and Independence as the Guiding Values for Future Research

During the concluding part of the quasi-focus group, when asked about the most critical aspect to consider when designing AALT houses, virtually all participants stressed the significance of dignity. They emphasized that technology should not diminish their self-worth and should be affordable. OA9 expressed concerns about financial accessibility, advocating for widely available technologies that enable individuals to maintain their dignity and independence and to be treated with respect. OA8 echoed this sentiment, emphasizing affordability to ensure everyone has a fair opportunity to preserve their dignity, be respected, and maintain independence. OA12 added to the discussion by emphasizing that technological advancements should primarily target OAs still capable of independent functioning. OA12 shared their experience of working with patients with Alzheimer disease for 15 years, recognizing the challenges they face in using complex devices due to their cognitive limitations and highlighted the need to develop gerontechnology adapted to people with Alzheimer disease that will be used primarily by their family caregivers to help them. However, they agreed with OA1 and OA10, highlighting the importance of assisting individuals who could benefit directly from some support rather than focusing solely on patients with Alzheimer disease.

### Role of OAs in Future Research

The concept of patient-partner emerged from the workshop discussion. OA10 emphasized the lack of involvement of older individuals in such research, according to her experience with different networks. This participant discussed the concept of the patient-partner in research, stating that she would like to be included in upcoming research projects as a patient-partner under ongoing partnership through videoconferencing and in-person attendance to co-design with researchers and help the researchers achieve progress effectively. According to the Canadian Institute of Health Research [33],

…if patients are involved in a research project in any manner other than as a research participant, they are considered “patient partners”. Some examples of the patient partner role may include participation on governing boards or committees, being consulted on survey design for a study, co-developing the research methodology with a researcher, taking part in priority-setting activities to determine new areas of research, and collecting and/or analyzing data and knowledge translation.

The concept of peer training or peer-to-peer support also emerged from the conversation. OA5 emphasized the need for expert guidance in using these technologies. While excited about having the latest devices at home, he acknowledged that OAs may struggle to navigate them independently. Having an expert to provide instruction and support was seen as highly beneficial. OA3 emphasized that although professional experts exist, it may be more convenient and cost-effective to develop online or in-person peer-training programs where “similar users” would help each other.

## Discussion

### Principal Findings

**Figure 2:**
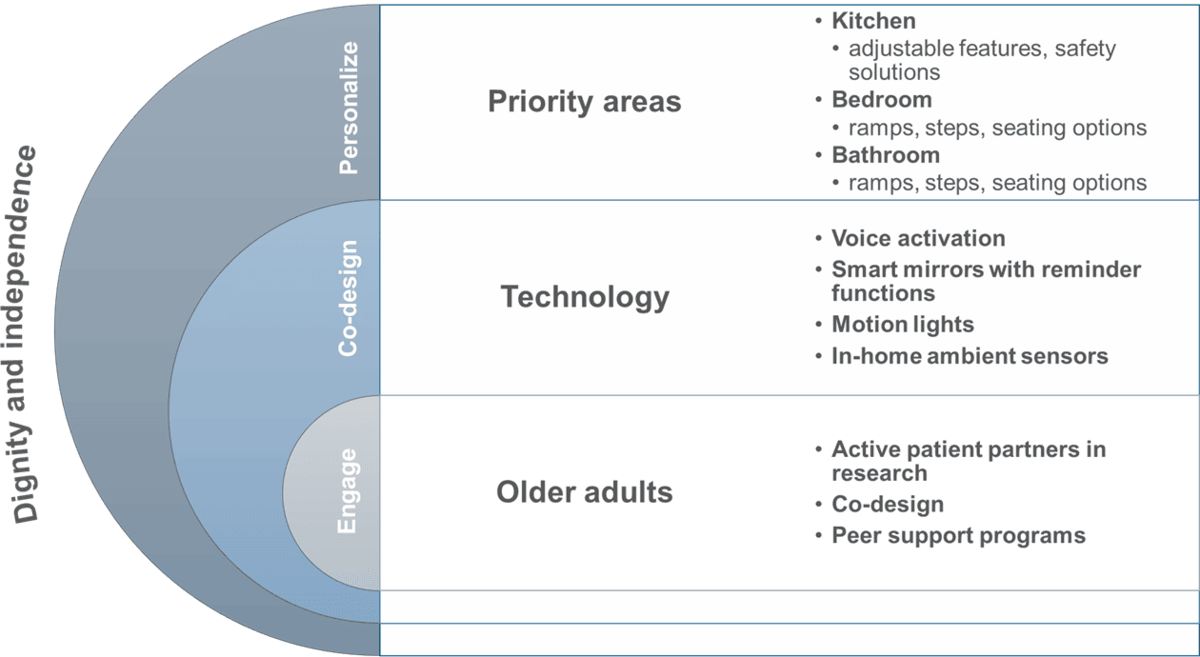
A roadmap for implementing gerontechnology to promote healthy aging in Manitoba, with a focus on priority areas such as engaging older adults, exploring technology, and co-designing personalized technology-based interventions.

This paper presents the outcomes of a coconstruction workshop conducted to tentatively draw a clear road map for developing gerontechnology in Manitoba (Figure 2) based on the ongoing investigator-led development of AALT. Overall, the workshop fostered positive interactions, and our participants appreciated the demonstrations and hands-on experience with the technology. Consequently, they actively engaged with both the researchers and each other, resulting in a collaborative workshop that generated new and practical concepts and ideas for research and development projects. There are growing attempts to develop AALT for gerontechnology applications worldwide; however, more tentative technology is available than established need-based and user-initiated programs involving gerontechnology, which is more likely because many tentative developments hastily designed a technology before establishing a needs assessment. Successful programs and technology implementation are more likely limited because of the uncertainty of designers about the most appropriate ways of translating the needs and priorities of OAs into technology for aging well. Digital ageism and marginalization of OAs may also have been reasons behind the unsuccessful gerontechnology development [34]. One of the design biases that researchers may fall into easily is failing to include OAs in technology development due to a suspected lack of technological abilities and the preconceptions accompanying this assumption. This study aimed to apprehend OAs’ perceptions, needs, and desires regarding gerontechnology and to explore better approaches to facilitate the inclusion of OAs in gerontechnology research and development. Our workshop progression exceeded our planned outcomes.

The first objective of this research work was to determine the best strategies for promoting aging well in the community with the support of gerontechnology. The key strategy identified by most of our workshop participants was the critical need for patient-designer partnerships from the beginning of the design process. Kitchen design emerged as a dominant theme in this discussion because it is considered by our participants a central area of a home where many activities that are key for autonomy are performed, and those activities are closely related to healthy living (eg, storing provisions and preparing meals). To the best of our knowledge, no research directly engaged OAs in cocreation activities. A recent workshop-based co-design study involved college students [35] to develop a 6-dimensional tentative model of the kitchen of the future was constructed with the potential to provide valuable insights for future development, including smart devices and interaction experiences, health and well-being, inclusivity and extensibility, ecosystem circulation and sustainability, emotional and meaningful experience, and spatial planning, and aesthetic experience [35]. Our research focused on the needs of OAs and is tentative to explicit the perceived health and wellness standards among OAs and operationalize these OAs’ needs into developmental actions. Most domestic injuries are related to working in the kitchen, and the OAs may have altered physical and cognitive capacities to use the kitchen as they used to during mid-adulthood. Accidents are more likely to happen among OAs depending on their balance, general fitness, and cognitive abilities. Accidents among OAs result in losing confidence in their abilities, reducing their self-esteem, and, in many circumstances, deciding to relocate to a nursing home. In our study, overall, participants stressed the importance of having a kitchen design that is accessible, safe, and accommodating for individuals with different abilities and mobility limitations, such as those observed among our research participants, namely, people with physical impairment, users of walkers, users of wheelchairs, and people with multiple sclerosis. The suggestions included modifications to the counter, the inclusion of stabilizers, and the provision of seating options, among others, to enhance usability and functionality in the kitchen space. The participants shared a common understanding that technology designed for OAs is meant to empower and enhance their quality of life while preserving their independence and dignity. They emphasized the importance of accessibility, affordability, and expert guidance to ensure OAs can navigate and benefit from these technologies without feeling diminished or excluded. These recommendations complement the literature on co-design among younger populations [35]. Interestingly, both studies focused more on the lifestyle and activities of the kitchen users rather than monitoring activity (passive data collection) to adapt the kitchen over time or prevent hazardous events such as falling in the kitchen or falling objects while using the kitchen area. There have been rising attempts to design monitoring systems for kitchen users [36-39] because the kitchen is the area of the house where many accidents occur, especially among OAs and those with dementia; however, these have not been highlighted by our research participants. The absence of discussion of advanced activity monitoring among our workshop participants may be related to a lack of awareness about ambient technology among lay populations, potential fear of the unknown future of the ethical handling of their data (when using video-based AALT [40]) or simple uninterest in advanced technology. Although these factors were not observed, we understand that our participants focused on their priority, namely a range of technology in the kitchen that supports accessibility, safety, and inclusion.

The second objective of this paper was to establish the top priorities for implementing gerontechnology in the community. The main strategies to promote aging well in the community that emerged from the workshop are to focus on OAs with memory impairments and finding ways to upgrade their homes with innovative technology. Our participants highlighted the need for reminders, motion detectors, and voice activation through robots and tablets to operate various household devices. These technologies offer a safer space for OAs with dementia or other cognitive impairments who may exhibit a higher risk of falls. For example, patients with dementia fall up to 8 times more often than those without dementia and have a lower recovery rate [41]. Patients with dementia typically need reminders, motion detectors and voice activation applications. A retrospective study using data from a general hospital’s electronic reporting system for inpatient falls reported that fallers with dementia were more likely to have vision impairment, urinary incontinence, use walking aids, and be confused and physically restrained at the time of the fall [42]. These facts encourage using ambient sensor systems in the bedroom, washroom, and kitchen with both day and night functionalities that allow monitoring activity to control for risks of falls and prevent falls and alert family members and care providers when a fall occurs. Reminders, motion detectors and voice activation have an important role in detection and monitoring mobility according to both our participants and the literature. A recent study, for example, examined an in-home monitoring system by 13 home-dwelling OAs, 13 family caregivers, and 20 nurses over the course of one year [43]. According to the majority of OA, family caregivers, and nurses, in-home sensors come as a second strategy and priority to promote aging well. Indeed, in-home sensors can help with staying at home, improving home care and quality of life, eliminating domestic mishaps, and reducing family stress [43]. Participants tended to be more in favor of ambient sensors than toward wearable technology. Nurses were less enthusiastic about in-home monitoring systems than OAs and their family caregivers expecting technology to weaken their relationship with OAs and lack of time to cover the needs of their patients while also handling the data generated from the in-home monitoring systems [43]. Surprisingly, physiological parameters (eg, heart rate, body temperature, and blood glucose levels) were less of a priority among our participants. There was less focus on physiological parameters, even though there is increasing evidence about the usefulness of ambient sensor systems in detecting health problems such as heart failure [44,45]. In-home monitoring systems appear feasible and well-accepted by users; however, more research is needed to raise awareness about the potential of in-home technology among end users (OAs, family caregivers, and health care providers) to take this field of exploration to the next level with more condition-specific studies that have the potential to demonstrate the likelihood of technology to contribute to OAs and assistive technology users’ independent living.

The third objective of this paper was to create a tentative road map for the creation and application of gerontechnology for aging well in the province of Manitoba based on the priorities and the strategies of the OAs aging in the province. The workshop enabled us to depict the guiding principles that must underlie our research, namely respecting dignity and independence as guiding values not only in servicing OAs but also in engaging them in the research decision-making processes and including OAs in all the research processes. There is a historic marginalization of OAs in technology development because of a perceived lack of technological skills and the stereotypes associated with this presumption [46]. The inclusion of OAs as central users of in-home technology and this principle would contribute to combatting digital health ageism [47], “self-ageism” [48], and should therefore be a guiding principle that spontaneously applies to any research and design of gerontechnology. There is a clear need for research on the inclusion of OAs in the research process centered on in-home technology. To the best of our knowledge, there is limited research on the engagement of OAs in the development of digital health solutions [49]. For example, the study by Kokorelias et al [49] examined the inclusion of OAs in hospital-to-home interventions and found inconsistency in the literature regarding the characteristics of the included participants because the OAs groups are not homogenous, implying the need for additional research to better understand how digital technologies to support hospital-to-home transitions can be inclusive [49]. However, there is a plethora of research on AAL usability which could be helpful to complement our proposed road map. AAL usability research has so far focused on presenting current trends and gaps in research, aiming to guide future developments in home health monitoring technologies [50-54], aligning AAL development with OAs’ preferences and needs [11,55-58] and evaluative approaches such as studying the strengths and limitations of the technology acceptance model in AALT research [6,59].

### Limitations

The workshop procedure likely primed participants to focus on the technologies showcased during the discovery phase, potentially biasing their responses toward the specific gerontechnologies demonstrated. Consequently, the authors acknowledge that these findings may not generalize to other gerontechnologies and recognize the possibility of unexplored needs, priorities, and potential benefits associated with alternative gerontechnologies that were not featured in the workshop.

Our workshop gathered people with physical impairment, users of walkers, users of wheelchairs, and people with multiple sclerosis; however, it may not be representative of other profiles who may need the technology presented in this paper, such as people with other chronic conditions, namely arthritis, epilepsy, chronic fatigue, diabetes, asthma, and high blood pressure. OAs are not a homogenous group, so the proposed road map and guiding principles are a pragmatic starting point for framework development that needs more exploration and coupling with in-home technology testing trials.

### Conclusion

This paper proposes a road map developed based on the needs of older Manitobans and their families gathered after a workshop activity with 14 OAs. The road map shows where OAs in Manitoba want development in their homes. It also says we should involve them in all research stages to make sure they’re part of the process of bringing new in-home ambient technology to life. The development presented in this paper is based on individual expertise and does not include OAs directly in conducting research or investing in infrastructure and equipment. The presented workshop outcomes highlighted this gap and the need to include OAs in all research steps to mature research and reach implementation stages. The presented outcomes have the potential to accelerate innovation in Manitoba and Canada and inspire medical and health sciences researchers to build capacity around aging-in-place and prioritize their work based on actual and pragmatic needs and wants. Further pragmatic research is needed to directly help increase the quality of life of OAs and their caregivers by research and implementation of in-home digital technologies in various contexts and for various conditions.

## Data Availability

Requests for access to data should be directed to the corresponding author and are subject to ethical approval for data sharing.

## Abbreviations

AALT: ambient assisting living technology
COREQ: consolidated criteria for reporting qualitative research
OA: older adult
VR: virtual reality
